# Supplementary material for: A Critical View of the Application of the APEX Software (Aqueous Photochemistry of Environmentally-Occurring Xenobiotics) to Predict Photoreaction Kinetics in Surface Freshwaters
Source: Molecules. 2019 Dec 18;25(1):9. doi: 10.3390/molecules25010009 (PMC7017383; doi:10.3390/molecules25010009)
Supplement: Supplementary file 1 [file molecules-25-00009-s001.zip › 3.2.4_gcc-4.4.0.zip!/3.2.4_gcc-4.4.0/3.2.4_gcc-4.4.0/doc/octave/html/interpreter/Commands.html]

Commands - Untitled


Next: Organization of Functions,
Previous: Function Handles Inline Functions and Anonymous Functions,
Up: Functions and Scripts


---

### 11.10 Commands

Commands are a special class of functions that only accept string
input arguments. A command can be called as an ordinary function, but
it can also be called without the parentheses like the following example
shows

```
     my_command hello world
```

which is the same as

```
     my_command("hello", "world")
```

The general form of a command call is

```
     name arg1 arg2 ...
```

which translates directly to

```
     name ("arg1", "arg2", ...)
```

A function can be used as a command if it accepts string input arguments.
To do this, the function must be marked as a command, which can be done
with the `mark_as_command` command like this

```
     mark_as_command name
```

where `name` is the function to be marked as a command.

One difficulty of commands occurs when one of the string input arguments
are stored in a variable. Since Octave can't tell the difference between
a variable name, and an ordinary string, it is not possible to pass a
variable as input to a command. In such a situation a command must be
called as a function.

— Built-in Function: **mark\_as\_command** (name)  
> This function is obsolete and will be removed from a future
> version of Octave.

— Built-in Function: **unmark\_command** (name)  
> This function is obsolete and will be removed from a future
> version of Octave.

— Built-in Function: **iscommand** (name)  
> This function is obsolete and will be removed from a future
> version of Octave.

— Built-in Function: **mark\_as\_rawcommand** (name)  
> This function is obsolete and will be removed from a future
> version of Octave.

— Built-in Function: **unmark\_rawcommand** (name)  
> This function is obsolete and will be removed from a future
> version of Octave.

— Built-in Function: **israwcommand** (name)  
> This function is obsolete and will be removed from a future
> version of Octave.
